# (4*S*)-Benzyl 4-isopropyl-5-oxo-1,3-oxazolidine-3-carboxyl­ate

**DOI:** 10.1107/S1600536808004455

**Published:** 2008-03-12

**Authors:** Jian-Bin Wu, Kan Lin, Jian-Nan Guo, Guo Tang, Yu-Fen Zhao

**Affiliations:** a Key Laboratory for Chemical Biology of Fujian Province, Department of Chemistry, Xiamen University, Xiamen 361005, People’s Republic of China

## Abstract

In the crystal structure of the title compound, C_14_H_17_NO_4_, obtained by the reaction of *N*-benzoxycarbonyl-l-valine, paraformaldehyde and 4-methyl­benzene­sulfonic acid, mol­ecules are linked by C—H⋯O hydrogen bonds, generating linear chains parallel to the *a* axis. C—H⋯π inter­actions of stacked benzene rings also provide stability for the crystal structure.

## Related literature

For related literature, see: Dorow & Gingrich (1999 ▶); Allen *et al.* (1987 ▶); Pavel *et al.* (1993 ▶); Reddy *et al.* (2000 ▶).
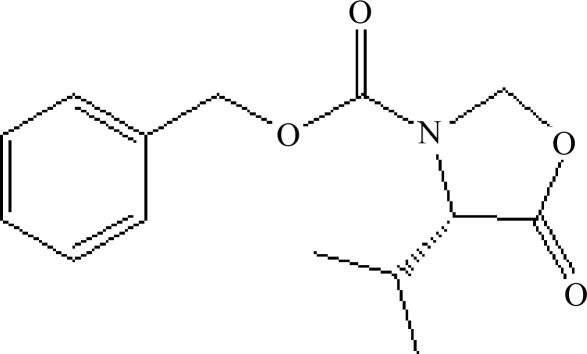


## Experimental

### 

#### Crystal data

C_14_H_17_NO_4_
*M* *_r_* = 263.29
Orthorhombic, 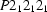

*a* = 6.0528 (2) Å
*b* = 13.1581 (5) Å
*c* = 16.6778 (6) Å
*V* = 1328.28 (8) Å^3^
*Z* = 4
Mo *K*α radiation
μ = 0.10 mm^−1^
*T* = 153 (2) K
0.50 × 0.17 × 0.09 mm

#### Data collection

Bruker APEX CCD diffractometer
Absorption correction: multi-scan (*SADABS*; Bruker, 2001 ▶) *T*_min_ = 0.953, *T*_max_ = 0.991
5718 measured reflections
1368 independent reflections
1100 reflections with *I* > 2σ(*I*)
*R*_int_ = 0.028

#### Refinement

*R*[*F* ^2^ > 2σ(*F* ^2^)] = 0.028
*wR*(*F* ^2^) = 0.062
*S* = 0.99
1368 reflections
172 parameters
H-atom parameters constrained
Δρ_max_ = 0.12 e Å^−3^
Δρ_min_ = −0.13 e Å^−3^

### 

Data collection: *SMART* (Bruker, 2001 ▶); cell refinement: *SAINT* (Bruker, 2001 ▶); data reduction: *SAINT*; program(s) used to solve structure: *SHELXS97* (Sheldrick, 2008 ▶); program(s) used to refine structure: *SHELXL97* (Sheldrick, 2008 ▶); molecular graphics: *ORTEP-3* (Farrugia, 1997 ▶); software used to prepare material for publication: *SHELXL97*.

## Supplementary Material

Crystal structure: contains datablocks I, global. DOI: 10.1107/S1600536808004455/cf2183sup1.cif

Structure factors: contains datablocks I. DOI: 10.1107/S1600536808004455/cf2183Isup2.hkl

Additional supplementary materials:  crystallographic information; 3D view; checkCIF report

## Figures and Tables

**Table 1:** Hydrogen-bond geometry (Å, °) *Cg* is the centroid of the C9–C14 benzene ring.

*D*—H⋯*A*	*D*—H	H⋯*A*	*D*⋯*A*	*D*—H⋯*A*
C2—H2*A*⋯O5^i^	0.97	2.32	3.258 (2)	163
C12—H12*A*⋯*Cg*^ii^	0.93	3.37	3.963 (3)	124

Symmetry codes: (i) 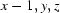; (ii) 
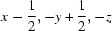
.

**Fig. 1.**
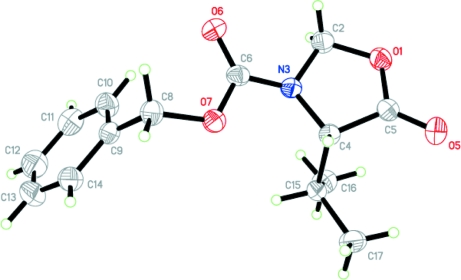
The molecular structure of (I) with the atom-labeling scheme, showing 50% probability displacement ellipsoids. H atoms are drawn as spheres of arbitrary radius.

**Fig. 2.**
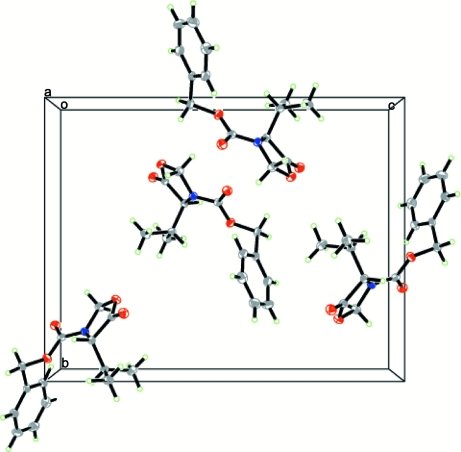
The packing of the molecules, viewed down the a axis.

**Fig. 3.**
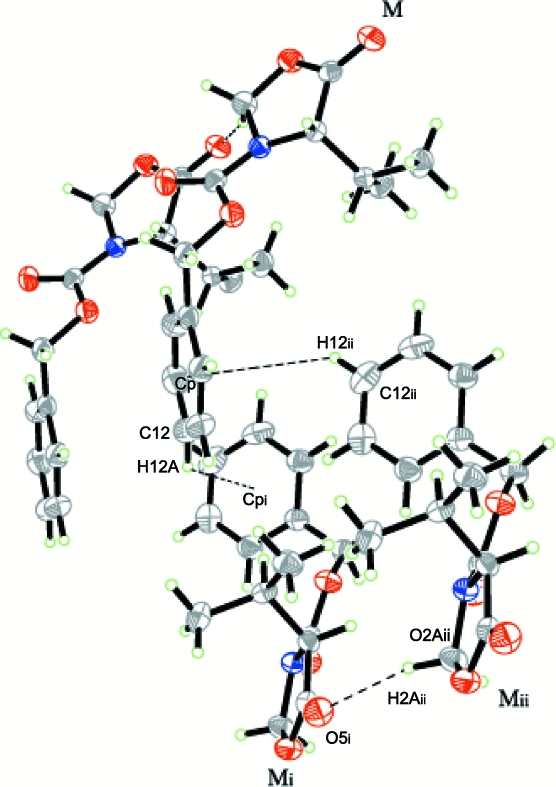
C—H···π interactions of (I). These and hydrogen bonds are shown as dashed lines. [Symmetry codes: (i) x - 1/2, -y + 1/2, -z; (ii) x + 1/2, -y + 1/2, -z.]

## supplementary crystallographic information

### Comment

The title compound (I) belongs to a class of 5-oxazolidinone and has been used to synthesize dipeptides and a series of biologically active molecules (Dorow & Gingrich, 1999).

In the compound, the oxazolidine ring is formed by the reaction of *N*-benzoxycarbonyl-*L*-valine, paraformaldehyde, and 4-methylbenzenesulfonic acid. The phenyl and the oxazolidine rings make a dihedral angle of 49.7 (1) (Fig. 1). The absolute configuration (S) of the stereocentre C4 remains unchanged during the synthetic procedure. An X-ray crystal structure determination of the molecular structure of compound (I) was carried out to determine its conformation. The bond lengths are within normal ranges (Allen *et al.*, 1987).

The packing is shown in Fig. 2. The occurrence of C—H···O hydrogen bond interactions lead to the formation of linear chains parallel to the *a* axis. The packing is further stabilized by C—H···π interactions of stacked benzene rings in the chains (Fig. 3), with typical geometry (Pavel *et al.*, 1993).

### Experimental

The title compound was prepared by a method based on one described by Reddy *et al.* (2000). A mixture of *N*-benzoxycarbonyl-*L*-valine (7.53 g, 3 mmol), paraformaldehyde (1.8 g, 6 mmol) and 4-methylbenzenesulfonic acid (PTSA, 0.31 g, 1.8 mmol) in benzene (25 ml) was refluxed, using a Dean–Stark apparatus, for about 1 h. After cooling, the resulting mixture was washed with 0.3 *M* aqueous K_2_CO_3_ solution (30 ml) followed by saturated aqueous NaCl solution (30 ml). The organic layer was separated and dried with Mg_2_SO_4_, filtered and concentrated *in vacuo* to give the crude product as a white solid (5.12 g, 65%). Crystals suitable for X-ray diffraction were obtained from an ethanol solution.

### Refinement

The hydrogen atoms were positioned geometrically (C—H = 0.93, 0.98, 0.97 or 0.96 Å for phenyl, tertiary, methylene or methyl H atoms respectively) and were included in the refinement in the riding model approximation. The displacement parameters of methyl H atoms were set to 1.5*U*_eq_(C), while those of other H atoms were set to 1.2*U*_eq_(C).

### Crystal data

**Table:**

C_14_H_17_NO_4_	*F*_000_ = 560
*M**_r_* = 263.29	*D*_x_ = 1.317 Mg m^−^^3^
Orthorhombic, *P*2_1_2_1_2_1_	Mo *K*α radiation λ = 0.71073 Å
Hall symbol: P 2ac 2ab	Cell parameters from 2839 reflections
*a* = 6.0528 (2) Å	θ = 2.9–32.6º
*b* = 13.1581 (5) Å	µ = 0.10 mm^−^^1^
*c* = 16.6778 (6) Å	*T* = 153 (2) K
*V* = 1328.28 (8) Å^3^	Needle, colourless
*Z* = 4	0.50 × 0.17 × 0.09 mm

### Data collection

**Table:**

Bruker APEX CCD diffractometer	1368 independent reflections
Radiation source: sealed tube	1100 reflections with *I* > 2σ(*I*)
Monochromator: graphite	*R*_int_ = 0.028
Detector resolution: 16.1903 pixels mm^-1^	θ_max_ = 25.0º
*T* = 153(2) K	θ_min_ = 2.9º
φ and ω scans	*h* = −7→7
Absorption correction: multi-scan(SADABS; Bruker, 2001)	*k* = −14→15
*T*_min_ = 0.953, *T*_max_ = 0.991	*l* = −19→19
5718 measured reflections	

### Refinement

**Table:**

Refinement on *F*^2^	Secondary atom site location: difference Fourier map
Least-squares matrix: full	Hydrogen site location: inferred from neighbouring sites
*R*[*F*^2^ > 2σ(*F*^2^)] = 0.028	H-atom parameters constrained
*wR*(*F*^2^) = 0.062	*w* = 1/[σ^2^(*F*_o_^2^) + (0.0398*P*)^2^] where *P* = (*F*_o_^2^ + 2*F*_c_^2^)/3
*S* = 0.99	(Δ/σ)_max_ < 0.001
1368 reflections	Δρ_max_ = 0.12 e Å^−^^3^
172 parameters	Δρ_min_ = −0.13 e Å^−^^3^
Primary atom site location: structure-invariant direct methods	Extinction correction: none

### Special details

**Table:**

Geometry. All e.s.d.'s (except the e.s.d. in the dihedral angle between two l.s. planes) are estimated using the full covariance matrix. The cell e.s.d.'s are taken into account individually in the estimation of e.s.d.'s in distances, angles and torsion angles; correlations between e.s.d.'s in cell parameters are only used when they are defined by crystal symmetry. An approximate (isotropic) treatment of cell e.s.d.'s is used for estimating e.s.d.'s involving l.s. planes.
Refinement. Refinement of *F*^2^ against ALL reflections. The weighted *R*-factor *wR* and goodness of fit *S* are based on *F*^2^, conventional *R*-factors *R* are based on *F*, with *F* set to zero for negative *F*^2^. The threshold expression of *F*^2^ > σ(*F*^2^) is used only for calculating *R*-factors(gt) *etc*. and is not relevant to the choice of reflections for refinement. *R*-factors based on *F*^2^ are statistically about twice as large as those based on *F*, and *R*- factors based on ALL data will be even larger.

### Fractional atomic coordinates and isotropic or equivalent isotropic displacement parameters (Å^2^)

**Table:**

	*x*	*y*	*z*	*U*_iso_*/*U*_eq_	
O1	−0.4445 (2)	−0.27913 (11)	0.18553 (8)	0.0312 (3)	
C2	−0.6151 (3)	−0.24832 (15)	0.13165 (12)	0.0315 (5)	
H2A	−0.7493	−0.2318	0.1607	0.038*	
H2B	−0.6473	−0.3017	0.0932	0.038*	
N3	−0.5269 (3)	−0.15900 (11)	0.09160 (9)	0.0250 (4)	
C4	−0.3046 (3)	−0.13532 (13)	0.11905 (11)	0.0242 (4)	
H4A	−0.2008	−0.1412	0.0742	0.029*	
O5	−0.1002 (2)	−0.23774 (11)	0.21593 (8)	0.0361 (4)	
C5	−0.2637 (3)	−0.22044 (15)	0.17824 (11)	0.0261 (4)	
O6	−0.7800 (2)	−0.17466 (10)	−0.00699 (9)	0.0348 (4)	
C6	−0.6135 (3)	−0.13483 (15)	0.01957 (12)	0.0268 (5)	
O7	−0.4976 (2)	−0.06146 (10)	−0.01706 (7)	0.0308 (3)	
C8	−0.5807 (4)	−0.02766 (16)	−0.09484 (11)	0.0325 (5)	
H8A	−0.6570	−0.0837	−0.1206	0.039*	
H8B	−0.4566	−0.0089	−0.1286	0.039*	
C9	−0.7346 (4)	0.06079 (15)	−0.08858 (11)	0.0287 (5)	
C10	−0.9408 (4)	0.05173 (17)	−0.05121 (12)	0.0361 (5)	
H10A	−0.9819	−0.0099	−0.0283	0.043*	
C11	−1.0833 (4)	0.13326 (18)	−0.04812 (13)	0.0435 (6)	
H11A	−1.2181	0.1269	−0.0218	0.052*	
C12	−1.0276 (4)	0.22421 (19)	−0.08374 (14)	0.0482 (6)	
H12A	−1.1247	0.2790	−0.0821	0.058*	
C13	−0.8260 (4)	0.23316 (17)	−0.12188 (15)	0.0467 (6)	
H13A	−0.7882	0.2941	−0.1466	0.056*	
C14	−0.6801 (4)	0.15252 (15)	−0.12363 (12)	0.0365 (5)	
H14A	−0.5438	0.1600	−0.1487	0.044*	
C15	−0.2829 (3)	−0.02961 (14)	0.15749 (11)	0.0266 (5)	
H15A	−0.3376	0.0202	0.1186	0.032*	
C16	−0.4247 (4)	−0.02032 (17)	0.23247 (13)	0.0378 (5)	
H16A	−0.4075	0.0464	0.2550	0.057*	
H16B	−0.3791	−0.0703	0.2710	0.057*	
H16C	−0.5769	−0.0312	0.2187	0.057*	
C17	−0.0424 (3)	−0.00377 (16)	0.17484 (13)	0.0375 (5)	
H17A	−0.0338	0.0625	0.1988	0.056*	
H17B	0.0401	−0.0043	0.1257	0.056*	
H17C	0.0182	−0.0532	0.2110	0.056*	

### Atomic displacement parameters (Å^2^)

**Table:**

	*U*^11^	*U*^22^	*U*^33^	*U*^12^	*U*^13^	*U*^23^
O1	0.0307 (8)	0.0288 (7)	0.0341 (7)	−0.0021 (7)	0.0004 (6)	0.0064 (6)
C2	0.0256 (11)	0.0325 (12)	0.0363 (11)	−0.0034 (10)	0.0005 (9)	0.0050 (10)
N3	0.0244 (9)	0.0243 (8)	0.0261 (9)	−0.0028 (7)	−0.0006 (7)	0.0013 (7)
C4	0.0224 (10)	0.0257 (10)	0.0244 (10)	−0.0005 (9)	0.0020 (8)	0.0008 (9)
O5	0.0331 (8)	0.0374 (8)	0.0378 (8)	0.0059 (7)	−0.0048 (7)	0.0036 (7)
C5	0.0274 (11)	0.0248 (10)	0.0261 (10)	0.0032 (9)	0.0033 (10)	−0.0028 (9)
O6	0.0328 (8)	0.0344 (8)	0.0372 (8)	−0.0060 (7)	−0.0088 (7)	−0.0012 (7)
C6	0.0267 (11)	0.0232 (11)	0.0304 (11)	0.0002 (10)	0.0014 (9)	−0.0039 (9)
O7	0.0325 (8)	0.0313 (7)	0.0286 (7)	−0.0057 (7)	−0.0029 (6)	0.0055 (6)
C8	0.0396 (12)	0.0346 (11)	0.0232 (10)	−0.0033 (10)	−0.0023 (9)	0.0027 (9)
C9	0.0375 (12)	0.0298 (11)	0.0187 (9)	−0.0039 (10)	−0.0045 (9)	0.0003 (9)
C10	0.0414 (13)	0.0358 (12)	0.0310 (11)	−0.0023 (11)	0.0010 (10)	0.0037 (10)
C11	0.0388 (13)	0.0509 (15)	0.0408 (13)	0.0027 (13)	−0.0022 (11)	−0.0038 (12)
C12	0.0554 (17)	0.0377 (14)	0.0514 (14)	0.0107 (13)	−0.0094 (13)	−0.0075 (12)
C13	0.0624 (16)	0.0283 (12)	0.0493 (13)	−0.0039 (12)	−0.0102 (13)	0.0045 (12)
C14	0.0430 (13)	0.0360 (12)	0.0305 (11)	−0.0070 (11)	−0.0022 (10)	0.0017 (11)
C15	0.0299 (11)	0.0212 (10)	0.0288 (10)	0.0002 (9)	−0.0049 (9)	0.0012 (8)
C16	0.0353 (12)	0.0364 (12)	0.0419 (12)	0.0045 (10)	0.0002 (10)	−0.0116 (10)
C17	0.0365 (13)	0.0365 (13)	0.0395 (12)	−0.0090 (11)	−0.0019 (10)	−0.0004 (10)

### Geometric parameters (Å, °)

**Table:**

O1—C5	1.345 (2)	C10—C11	1.378 (3)
O1—C2	1.428 (2)	C10—H10A	0.930
C2—N3	1.454 (2)	C11—C12	1.378 (4)
C2—H2A	0.970	C11—H11A	0.930
C2—H2B	0.970	C12—C13	1.381 (4)
N3—C6	1.349 (2)	C12—H12A	0.930
N3—C4	1.455 (2)	C13—C14	1.381 (3)
C4—C5	1.513 (3)	C13—H13A	0.930
C4—C15	1.537 (2)	C14—H14A	0.930
C4—H4A	0.980	C15—C16	1.522 (3)
O5—C5	1.194 (2)	C15—C17	1.523 (3)
O6—C6	1.219 (2)	C15—H15A	0.980
C6—O7	1.340 (2)	C16—H16A	0.960
O7—C8	1.461 (2)	C16—H16B	0.960
C8—C9	1.495 (3)	C16—H16C	0.960
C8—H8A	0.970	C17—H17A	0.960
C8—H8B	0.970	C17—H17B	0.960
C9—C14	1.381 (3)	C17—H17C	0.960
C9—C10	1.400 (3)		
			
C5—O1—C2	111.63 (15)	C11—C10—H10A	119.7
O1—C2—N3	104.66 (15)	C9—C10—H10A	119.7
O1—C2—H2A	110.8	C10—C11—C12	120.5 (2)
N3—C2—H2A	110.8	C10—C11—H11A	119.8
O1—C2—H2B	110.8	C12—C11—H11A	119.8
N3—C2—H2B	110.8	C11—C12—C13	119.3 (2)
H2A—C2—H2B	108.9	C11—C12—H12A	120.4
C6—N3—C2	117.21 (16)	C13—C12—H12A	120.4
C6—N3—C4	126.09 (16)	C14—C13—C12	120.6 (2)
C2—N3—C4	111.61 (15)	C14—C13—H13A	119.7
N3—C4—C5	101.42 (15)	C12—C13—H13A	119.7
N3—C4—C15	113.81 (15)	C13—C14—C9	120.7 (2)
C5—C4—C15	112.56 (15)	C13—C14—H14A	119.7
N3—C4—H4A	109.6	C9—C14—H14A	119.7
C5—C4—H4A	109.6	C16—C15—C17	111.42 (17)
C15—C4—H4A	109.6	C16—C15—C4	111.55 (16)
O5—C5—O1	121.14 (18)	C17—C15—C4	111.28 (16)
O5—C5—C4	128.30 (19)	C16—C15—H15A	107.4
O1—C5—C4	110.55 (16)	C17—C15—H15A	107.4
O6—C6—O7	125.19 (18)	C4—C15—H15A	107.4
O6—C6—N3	122.92 (18)	C15—C16—H16A	109.5
O7—C6—N3	111.88 (17)	C15—C16—H16B	109.5
C6—O7—C8	116.37 (15)	H16A—C16—H16B	109.5
O7—C8—C9	112.93 (16)	C15—C16—H16C	109.5
O7—C8—H8A	109.0	H16A—C16—H16C	109.5
C9—C8—H8A	109.0	H16B—C16—H16C	109.5
O7—C8—H8B	109.0	C15—C17—H17A	109.5
C9—C8—H8B	109.0	C15—C17—H17B	109.5
H8A—C8—H8B	107.8	H17A—C17—H17B	109.5
C14—C9—C10	118.4 (2)	C15—C17—H17C	109.5
C14—C9—C8	120.14 (19)	H17A—C17—H17C	109.5
C10—C9—C8	121.37 (18)	H17B—C17—H17C	109.5
C11—C10—C9	120.6 (2)		
			
C5—O1—C2—N3	−3.0 (2)	O6—C6—O7—C8	0.1 (3)
O1—C2—N3—C6	157.15 (15)	N3—C6—O7—C8	−178.52 (15)
O1—C2—N3—C4	0.8 (2)	C6—O7—C8—C9	92.1 (2)
C6—N3—C4—C5	−152.48 (17)	O7—C8—C9—C14	117.2 (2)
C2—N3—C4—C5	1.34 (19)	O7—C8—C9—C10	−66.2 (2)
C6—N3—C4—C15	86.4 (2)	C14—C9—C10—C11	−1.4 (3)
C2—N3—C4—C15	−119.80 (17)	C8—C9—C10—C11	−178.02 (19)
C2—O1—C5—O5	−176.54 (18)	C9—C10—C11—C12	1.8 (3)
C2—O1—C5—C4	4.0 (2)	C10—C11—C12—C13	−0.7 (4)
N3—C4—C5—O5	177.38 (19)	C11—C12—C13—C14	−0.8 (4)
C15—C4—C5—O5	−60.6 (3)	C12—C13—C14—C9	1.2 (3)
N3—C4—C5—O1	−3.20 (19)	C10—C9—C14—C13	−0.1 (3)
C15—C4—C5—O1	118.80 (17)	C8—C9—C14—C13	176.57 (19)
C2—N3—C6—O6	11.0 (3)	N3—C4—C15—C16	62.4 (2)
C4—N3—C6—O6	163.56 (17)	C5—C4—C15—C16	−52.3 (2)
C2—N3—C6—O7	−170.34 (15)	N3—C4—C15—C17	−172.49 (16)
C4—N3—C6—O7	−17.8 (2)	C5—C4—C15—C17	72.8 (2)

### Hydrogen-bond geometry (Å, °)

**Table:**

*D*—H···*A*	*D*—H	H···*A*	*D*···*A*	*D*—H···*A*
C2—H2A···O5^i^	0.97	2.32	3.258 (2)	163
C12—H12A···Cg^ii^	0.93	3.37	3.963 (3)	124

Symmetry codes: (i) *x*−1, *y*, *z*; (ii) *x*−1/2, −*y*+1/2, −*z*.
